# The Efficacy of Vaginal Clindamycin for the Treatment of Abnormal Genital Tract Flora in Pregnancy

**DOI:** 10.1080/10647440300025519

**Published:** 2003

**Authors:** Ronald F. Lamont, Brian M. Jones, Debashis Mandal, Philip E. Hay, Marie Sheehan

**Affiliations:** 1 Northwick Park and St Mark's NHS Trust Harrow Middlesex United Kingdom; 2 University of Sheffield Sheffield United Kingdom; 3 Manchester Centre for Sexual Health Manchester Royal Infirmary Manchester United Kingdom; 4 St George's Hospital Tooting London SW17 United Kingdom; 5 Imperial College School of Science Technology and Medicine London United Kingdom; 6 Department of Obstetrics and Gynaecology Northwick Park and St Mark's NHS Trust Watford Road Harrow, Middlesex London HA1 3UJ United Kingdom

## Abstract

*Objective:* To assess the efficacy of 2% clindamycin vaginal cream (CVC) to treat bacterial vaginosis (BV) in pregnancy.

*Methods:* A prospective, randomized, double-blind, placebo-controlled, tricenter study. Four hundred and four women with BV on Gram stain at their first antenatal clinic visit were randomized to receive a 3-day course of 2% CVC or placebo. The outcome was assessed using an intention to treat analysis at 3 weeks and 6 weeks post-treatment according to three different diagnostic methods based on five criteria (Gram stain and all four elements of clinical composite criteria: vaginal discharge, abnormal vaginal pH, clue cells, amine odor), three criteria (vaginal pH, clue cells, amine odor) or two criteria (clue cells and amine odor) to reflect stringency of diagnosis, historical precedence and government agency recommendations respectively.

*Results:* Using five diagnostic criteria, 18% of CVC patients were cured and 70.8% either cured and/or improved compared to 1.6% and 12% of placebo patients respectively (*p* < 0.0001). Using three diagnostic criteria, 44.8% of CVC patients were cured and 77.3% were either cured and/or improved compared to 9.3% and 28.8% of placebo patients respectively (*p* < 0.0001). Using two diagnostic criteria, 75.0% of CVC patients were cured compared to 18.0% of placebo patients (p < 0.0001). Recurrence rates in those CVC patients successfully treated were approximately 6% at 6 weeks post baseline and 10% at 28 to 34 weeks gestation.

*Conclusions:*  A 3-day course of CVC appears to be well tolerated by the mother and statistically significantly more efficacious than placebo in the treatment of BV during the second trimester of pregnancy.


*Infect Dis Obstet Gynecol 2003;11:181–189*

### The efficacy of vaginal clindamycin for the treatment of abnormal genital tract flora in pregnancy

**Ronald F. Lamont[1,5], Brian M. Jones[2], Debashis Mandal[3], Philip E. Hay[4] and Marie Sheehan[5]**

[1]*Northwick Park and St Mark's NHS Trust, Harrow, Middlesex, UK*
[2]*University of Sheffield, Sheffield, UK*
[3]*Manchester Centre for Sexual Health, Manchester Royal Infirmary, Manchester, UK*
[4]*St George's Hospital, Tooting, London SW17, UK*
[5]*Imperial College School of Science, Technology and Medicine, London, UK*

***Objective:*** To assess the efficacy of 2% clindamycin vaginal cream (CVC) to treat bacterial vaginosis (BV) in pregnancy.
***Methods:*** A prospective, randomized, double-blind, placebo-controlled, tricenter study. Four hundred and four women with BV on Gram stain at their first antenatal clinic visit were randomized to receive a 3-day course of 2% CVC or placebo. The outcome was assessed using an intention to treat analysis at 3 weeks and 6 weeks post-treatment according to three different diagnostic methods based on five criteria (Gram stain and all four elements of clinical composite criteria: vaginal discharge, abnormal vaginal pH, clue cells, amine odor), three criteria (vaginal pH, clue cells, amine odor) or two criteria (clue cells and amine odor) to reflect stringency of diagnosis, historical precedence and government agency recommendations respectively.
***Results:*** Using five diagnostic criteria, 18% of CVC patients were cured and 70.8% either cured and/or improved compared to 1.6% and 12% of placebo patients respectively ($p < 0.0001$). Using three diagnostic criteria, 44.8% of CVC patients were cured and 77.3% were either cured and/or improved compared to 9.3% and 28.8% of placebo patients respectively ($p < 0.0001$). Using two diagnostic criteria, 75.0% of CVC patients were cured compared to 18.0% of placebo patients ($p < 0.0001$). Recurrence rates in those CVC patients successfully treated were approximately 6% at 6 weeks post baseline and 10% at 28 to 34 weeks gestation.
***Conclusions:*** A 3-day course of CVC appears to be well tolerated by the mother and statistically significantly more efficacious than placebo in the treatment of BV during the second trimester of pregnancy.

Key words: CLINDAMYCIN; PREGNANCY; EFFICACY; BACTERIAL VAGINOSIS

Bacterial vaginosis (BV) is a common poly-microbial condition previously known by a variety of names. It is associated with a reduction in the number and quality of lactobacilli, together with a 1000-fold increase in other organisms, such as anaerobes, *Mycoplasma hominis* and *Gardnerella vaginalis*. These changes in the vaginal flora are reflected in the appearance of a Gram stain of vaginal secretions and form the basis of diagnosis[1,2]. The disease is detected clinically by the observation of a set of signs first described by Amsel and co-workers in 1983 and adopted as the common clinical criteria for diagnosis and assessment of treatment[3]. A number of studies have shown that

Correspondence to: Mr R. F. Lamont, Department of Obstetrics and Gynaecology, Northwick Park and St Mark's NHS Trust, Watford Road, Harrow, Middlesex HA1 3UJ, London, UK. Email: pauline.mills@nwlh.nhs.uk

© 2003 The Parthenon Publishing Group

BV is associated with a two- to five-fold increased risk of preterm birth which is the greatest cause of perinatal mortality and morbidity in the developed world. The earlier in pregnancy at which BV is diagnosed, the greater is the relative risk of adverse outcome[4]. For this reason and also because BV can cause a malodorous vaginal discharge, it may be necessary to treat BV in pregnancy. The Centers for Disease Control and Prevention (Atlanta, GA, USA) have recommended that all women who have symptomatic disease require treatment regardless of pregnancy status and that there is enough evidence of benefit from prophylaxis to reduce the incidence of preterm birth that high-risk pregnant women who do not have symptoms of BV may be evaluated for treatment[5]. To prevent adverse fetomaternal side-effects, it would be more appropriate to use local rather than systemic therapy. This study is unique in that it is the only study to have evaluated the efficacy and safety of a 3-day course of 2% intravaginal clindamycin cream for the treatment of BV in pregnancy.

### SUBJECTS AND METHODS

The study was a prospective, randomized block, double-blind, placebo-controlled, parallel study group carried out over a 3-year period. Inclusion criteria required women to be pregnant, asymptomatic, aged 16 to 40 years, with a live fetus between 13 and 20 weeks gestation at their first antenatal clinic visit and with a Gram stain of vaginal secretions consistent with a diagnosis of BV[1]. Women were excluded if they had a known sensitivity to clindamycin, a history of antibiotic-related colitis, inflammatory bowel disease or frequent periodic diarrhea or if they had a concomitant sexually transmitted disease (STD). During application of the cream, women were encouraged to abstain from sexual intercourse. Women were to self administer 5 g of intravaginal clindamycin cream (CVC) equivalent to 100 mg of clindamycin or placebo, at night for three consecutive nights before retiring to bed. If women had sexual intercourse they were advised to use a condom during the time of treatment. Treatments were assigned using a computerized, randomized block procedure with a block size of 10 using a computer-generated random code list. The investigational drug and the placebo cream were of identical composition except for the active drug which accounted for a small proportion of the overall formulation. Active and placebo creams were packaged identically and labeled only with protocol number, patient number, abbreviated instructions for use and an institution to return the part used or empty tube. The full blind treatment code was located in the pharmacology department of the study site in the form of the random code list while individual subject code envelopes were attached to the rear inside cover of the case report form.

### Schedule of efficacy measurements

Following their baseline first antenatal clinic visit at 13 to 20 weeks gestation (median 16 weeks), efficacy data were collected at visit 2 (20 to 24 days post baseline), visit 3 (20 to 24 days post visit 2) and visit 4 (28 to 24 weeks gestation).

Final data were collected 24 to 48 hours post delivery. At baseline screen, following eligibility check list and informed consent, a sterile Cusco's speculum was used to sample upper vaginal secretions. These secretions were examined using Gram stain for the diagnosis of BV[1]. Evidence of clue cells (desquamated epithelial cells whose margins are obscured by large numbers of coccobacilli), abnormal vaginal discharge, vaginal pH and release of amine odor on addition of 10% potassium hydroxide were also recorded. An endocervical swab and high vaginal swab was carried out to exclude STD with *Neisseria gonorrhoeae*, *Chlamydia trachomatis* or *Trichomonas vaginalis*. If the Gram stain showed evidence of BV, the patient was randomized to received CVC or placebo. At visits 2, 3 and 4 Gram stain and composite criteria were repeated. In the CVC group, 178 (89.4%), 160 (80.4%) and 153 (76.9%) attended visits 2, 3 and 4, respectively. This was similar to those women in the placebo group whose attendance was 190 (92.7%), 170 (82.9%) and 162 (79%), respectively.

### Statistical methods

All statistical methods were performed using an SAS software in a mainframe environment. Statistical tests were two-sided and *p*-values of less than 0.05 were deemed to be statistically significant. Continuous variables, such as age, height, past obstetric history, gestational age and Apgar scores were assumed to have continuous, symmetric distributions and were analyzed using two sample *t*-tests. Categorical variables, such as race, social history, baseline high vaginal swab, pregnancy outcome, resolution of BV and proportion of fetomaternal adverse effects were analyzed using Chi-squared tests. Fisher's exact test was used in cases where the expected cell frequency was less than five. Sample size was calculated on the basis of the probability of a power of at least 80% (correct positive) to detect the difference between CVC and placebo groups of at least 10% in the treatment of BV (cure or improvement) at visit 2 at a significance level of 5% (false positive).

### Measure of efficacy

The primary efficacy measure was the resolution of BV. At the time of preparation of the protocol, the 1998 Food and Drug Administration (FDA) Guidance for Studies evaluating the treatment of bacterial vaginosis had not been published. Only the FDA recommendations of 1990 were available and, as now, there was debate as to whether to use clinical criteria, Gram stain, a combination of the two or other methods of diagnosing BV. Accordingly, in order to maintain stringency of diagnosis and to conform to historical diagnostic methods and available drug agency recommendations at the time of study design and to allow comparison with other studies, the resolution of BV was determined using three distinct definitions of clinical outcome as follows:

(i) Based on five diagnostic criteria to reflect stringency of diagnosis (positive Gram stain, vaginal pH > 4.5, presence of clue cells, amine odor and characteristic vaginal discharge). Post-treatment, at visit 2, patients with resolution of these five diagnostic criteria were considered cured. Patients with a negative Gram stain with one or more of the remaining four clinical criteria unresolved were considered improved. Patients with a positive Gram stain (regardless of composite criteria) were considered failed.

(ii) Based on three diagnostic criteria used to reflect the historical criteria of Amsel and co-workers[3] (vaginal pH > 4.5, presence of clue cells and amine odor). Post-treatment, at visit 2, patients with resolution of all three diagnostic criteria were considered cured. Patients with any two criteria resolved were considered improved and patients with fewer than two criteria resolved were considered failed.

(iii) Based on two diagnostic criteria (presence of clue cells and amine odor), the United States FDA recommendation that efficacy data should be analyzed using these criteria were tested. The FDA based this recommendation on evidence that the presence of these two criteria more accurately predicted BV (99% accuracy) than vaginal fluid[6]. Post-treatment, at visit 2, patients with both diagnostic criteria resolved were considered cured and patients with either criteria unresolved were considered failed. The absence of adequate data to categorize an outcome as cured or failed were considered non-assessable.

In all the analyses of efficacy for the treatment of BV, the outcome was assessed at visit 2, though visit 3 was used to assess recurrence in those women successfully treated at visit 2. There was no center-to-center variation in the results of the evaluation so the group is considered as a whole. While the analysis is presented as an intention to treat analysis the observations of safety and efficacy were identical when those patients who failed to receive study drug or failed to receive study drug as per protocol were excluded.

A total of 404 women were studied in an intention to treat (ITT) analysis comprising 199 in the CVC group and 205 in the placebo group. There were no significant differences between the CVC and placebo groups with respect to age, weight, height, race, gestational age at baseline, history of smoking, alcohol or substance abuse (Table 1). Similarly, there was no significant difference between the groups with respect to past

**Table 1** Demographic characteristics and social history of all treated patients at study entry

| *Demographic variable* | *CVC* *n* = 199 | *Placebo* *n* = 205 | *Treatment p-value** |
|---|---|---|---|
| Mean age, yr ± SD | 27.2 ± 5.07 | 27.1 ± 5.05 | 0.7009 |
| [Range: number reporting] | [16–40; *n* = 198] | [16–40; *n* = 205] | |
| Mean weight, kg ± SD | 66.5 ± 13.42 | 64.2 ± 10.97 | 0.0654 |
| [Range: number reporting] | [40.0–131.5; *n* = 199] | [42–103; *n* = 203] | |
| Mean height, m ± SD | 1.64 ± 0.66 | 1.63 ± 0.64 | 0.1166 |
| [Range: number reporting] | [1.35–1.81; *n* = 199] | [1.46–1.82; *n* = 205] | |
| Race: | | | |
| White | 143 (71.9%) | 136 (66.3%) | |
| Black | 30 (15.1%) | 35 (17.1%) | 0.463 |
| Other | 26 (13.1%) | 34 (16.6%) | |
| *Type of history* | | | *p-value*** |
| Smoking | | | |
| Yes | 55 (27.6%) | 67 (32.7%) | |
| No | 144 (72.4%) | 138 (67.3%) | 0.2695 |
| Cigarettes smoked/day, mean ± SD | 2.5 ± 4.9 | 3.2 ± 6.3 | |
| Drinking alcohol | | | |
| Yes | 42 (21.1%) | 39 (19.0%) | |
| No | 157 (78.9%) | 166 (81.0%) | 0.6014 |
| Alcoholic drinks/week, mean ± SD | 0.5 ± 1.9 | 0.6 ± 1.7 | |
| Substance abuse | | | |
| Yes | 4 (2.0%) | 4 (2%) | 0.9661 |
| No | 195 (98.0%) | 201 (98%) | |

CVC, clindamycin vaginal cream. *For age, weight and height, based on two-sample F-test (excluding not reported); for race, based on Chi-squared test (based on white, black and all other races). Statistics are based on the number of patients reporting data. **Based on Chi-squared test of yes or no medical history, gravidity, parity or other factors in the past obstetric history or history of STD. In total, 224 women, 123 women and 57 women were recruited from Northwick Park Hospital, Jessop Hospital, Sheffield and Bolton General Hospital, respectively. There were no significant differences between the three centers so the pooled results of all three centers are presented.

### Safety measures

Safety was monitored by the reporting of maternal and neonatal medical events (ME) including (i) overall incidence of ME, (ii) overall incidence of drug-related ME, (iii) overall incidence of ME leading to dropout, (iv) overall incidence of serious ME and (v) incidence of maternal and neonatal death.

### RESULTS

Table 2 shows the treatment outcome at visit 2 based on five, three and two diagnostic criteria using an ITT analysis. With the five diagnostic (stringent) criteria for cure used in this analysis, cure rates were 18% for CVC patients and 1.6% for placebo patients. Improvement rates were 52.6% for CVC patients and 10.4% for placebo patients and combined cured and improved rates were 70.8% for CVC patients and 12.0% for placebo patients. Statistically significant differences between treatment groups ($p < 0.0001$) were observed for the distribution among cured, improved and failed, as well as for analyses combining outcomes. Using three diagnostic criteria, cure rates were considerable higher than those obtained for the analysis based on five diagnostic criteria, which follows from the less

**Table 2** Intention to treat analysis of the outcome at visit 2, based on five, three and two diagnostic criteria

| | *Number of patients (% of group)* | | | | | |
|---|---|---|---|---|---|---|
| | *Five diagnostic criteria* | | *Three diagnostic criteria* | | *Two diagnostic criteria* | |
| *Treatment outcome* | *CVC* $n = 178$ | *Placebo* $n = 190$ | *CVC* $n = 178$ | *Placebo* $n = 190$ | *CVC* $n = 178$ | *Placebo* $n = 190$ |
| Cured$^{+}$ | 31 (18)* | 3 (1.6)* | 77 (44.8)* | 17 (9.3)* | 129 (75.0)* | 33 (18.0)* |
| Improved$^{++}$ | 90 (52.6)* | 19 (10.4)* | 56 (32.6)* | 21 (11.5)* | N/A | N/A |
| Failed$^{\dagger}$ | 50 (29.2)* | 161 (88.0)* | 39 (22.7)* | 145 (79.2)* | 43 (25.0)* | 150 (82.0)* |
| Cured and improved | 121 (70.8)* | 22 (12.0)* | 133 (73.3)* | 38 (28.8)* | N/A | N/A |

CVC, clindamycin vaginal cream, *$p < 0.0001$ based on $\chi^2$ (placebo versus CVC treatment). $^{+}$For five criteria, negative Gram stain with resolution of pH, clue cells, amine odor and discharge; for three criteria, resolution of pH, clue cells and amine odor; for two criteria, resolution of both clue cells and amine odor. $^{++}$For five criteria, negative Gram stain with one or more other criteria unresolved; for three criteria, two of three resolved (pH, clue cells, amine odor). $^{\dagger}$For five criteria, positive Gram stain; for three criteria, one of three resolved (pH, clue cells, amine odor); for two criteria, clue cells or amine odor unresolved stringent definition of cure. Cure rates were 44.8% for CVC patients and 9.3% for placebo patients. Improvement rates were 32.6% for CVC patients and 11.5% for placebo patients and combined cured and improved were 77.3% for CVC patients and 28.8% for placebo patients. Statistically significant differences between treatment groups ($p < 0.0001$) were observed for all the separate and combined outcomes analyzed. Using two diagnostic criteria, cure rates were similar to the combined cured and improved rates for the analysis based on three diagnostic criteria. Statistically significant differences in outcome between treatment groups ($p < 0.0001$) were observed. Cure rates were 75.0% for CVC patients and 18.0% for placebo patients. Recurrence rates at visit 3 and 4 for those patients cured or improved at visit 2 (by five criteria) according to criteria-based diagnosis is shown in Table 3. For CVC patients, recurrence rates were approximately 6% at visit 3 and 10% at 28–34 weeks gestation. This is in contrast to approximately 20% and 25% for placebo patients, respectively.

### Maternal safety outcome

For CVC versus placebo patients, respectively, the overall incidence of ME (53.8% versus 47.8%), those ME leading to dropout (0.5% versus 1.5%) and serious ME (7.5% versus 10.2%) did not differ significantly. While there was an excess of 20

**Table 3** Intention to treat analysis of recurrence rate in CVC-treated patients at visits 3 and 4 for those cured or improved at visit 2

| *Diagnostic basis* | *Visit 3* | | *Visit 4* | |
|---|---|---|---|---|
| | *CVC* | *Placebo* | *CVC* | *Placebo* |
| 5-criteria | 6.3% | 15% | 10.3% | 23.8% |
| 3-criteria | 5.4% | 20% | 8.4% | 25% |
| 2-criteria | 7.1% | 25% | 11.2% | 25% |

CVC, clindamycin vaginal cream

(10.1%) versus 4 (2.0%) for those women with greater than or equal to one drug-related ME, this was due mainly to non-serious conditions such as vaginal moniliasis (5.5%) and pruritis (2.0%).

### Neonatal safety outcome

For CVC versus placebo patients, respectively, the overall incidence of ME (12.8% versus 9.5%) and drug-related ME (0% versus 0%) did not differ significantly.

There were no maternal deaths. Neonatal deaths and serious MEs were reported for a similar percentage of neonates delivered for CVC patients (deaths 1.5%; serious MEs 5.6%) and placebo patients (deaths 1.5%; serious MEs 5%). None of these events was reported to be related to study medication.

### DISCUSSION

This study has shown that by whatever criteria of efficacy, a 3-day course of CVC used in the second trimester of pregnancy is an effective way to treat abnormal genital tract flora manifest by bacterial vaginosis on Gram stain of vaginal secretions. The effect this treatment has on the outcome of pregnancy has been the subject of a separate report[7]. Clindamycin phosphate 2% vaginal cream has been shown to be effective in the treatment of non-pregnant women with BV[8,9]. A 7-day regime is approved for this indication in many countries. Clindamycin 2% cream is associated with few side-effects and only a small fraction (approximately 4%) is absorbed systemically[10]. This study is the largest study to examine the efficacy and safety of CVC in pregnancy and the only study to examine a 3-day course in pregnancy. In pregnant women, a 7-day course is effective[11]. In non-pregnant women a 3-day course was as effective as a 7-day course[12] and significantly better than placebo[13]. A 3-day course is likely to be much more acceptable than a 7-day course and therefore more likely to increase patient compliance. Historically, the diagnosis of BV has been based on three out of four clinical criteria according to the original study of Amsel and co-workers[3]. The use of composite criteria is clumsy, unpleasant and irreproducible and likely to be replaced by Gram stain, which has excellent specificity and sensitivity when measured against composite criteria[2]. In view of this, three diagnostic methods based on five, three and two criteria corresponding to a very stringent diagnosis including Gram stain, the Amsel classification[3] and the US FDA recommendation[6], respectively, were chosen to reflect the range of diagnostic criteria used at the time of study design. Using the current American FDA guidelines, the evaluation of the studies for the treatment of BV would correspond with the five criteria diagnostic method, a combination of Gram stain and all four clinical criteria. Some still feel that the Amsel clinical criteria should be the gold standard and at the time the study was conducted only the 1990 FDA recommendations were available and these suggested using two criteria. With all these diagnostic criteria, clindamycin vaginal cream 2% administered as a 5 g dose once nightly for three consecutive nights was found to be statistically significantly superior to placebo and effective in the treatment of BV during the second trimester of pregnancy. Of those patients in the five, three and two criteria diagnostic groups, 18%, 44.8% and 75.0% in the treatment group and 1.6%, 9.3% and 18.0% in the placebo group, respectively, were cured ($p < 0.0001$). In the five and three criteria diagnostic groups, 70.8% and 77.3% in the treatment group and 12.0% and 28.8% in the placebo group, respectively, showed either cure or improvement ($p < 0.0001$). With only two diagnostic criteria there was no acceptable definition for *improved* so this last group could not be included in a *cured* and *improved* category.

A cure rate of 44.8% based on three diagnostic criteria compared favorably with the 7-day unpublished study[11] where the corresponding cure rate was 50.8% using a less stringent definition of cure (resolution of any three out of four criteria). Relative to the 7-day unpublished study[11], a lower number of CVC patients in our study reported any medical events and the proportion of CVC patients in our study with events considered to be drug-related was about half of the earlier study. The treatment appears to be well tolerated in this patient population and was not considered to have caused any adverse neonatal or serious maternal events. Recurrence rates were low at approximately 6% at visit 3 and 10% at 28 to 34 weeks gestation.

Bacterial vaginosis is thought to account for up to one-third of vulvovaginal infectious morbidity and is associated with adverse sequelae in both obstetrics and gynaecology[14]. The symptoms relating to BV are said to account for 10 million clinic visits annually in the USA[15]. Prevalence rates vary according to population studied but in an unselected, antenatal population in a district general hospital in the UK, 15% of women at the first antenatal visit were found to have this condition[16]. Although up to half of these women with BV may be asymptomatic, women who are symptomatic may experience a distressing, malodorous, offensive vaginal discharge due to volatile amines released at high pH from their stable salt form at low pH. BV in pregnancy is associated with postpartum endometritis[17] and chorioamnionitis[18]. A large number of studies have shown an association between BV and preterm prelabor rupture of the membranes and preterm delivery and late miscarriage and these have recently been reviewed[19]. Kurki and co-workers found a 2.6-fold increased risk of preterm labor and a 6.9-fold increased risk of preterm delivery with BV in early pregnancy[20]. In South-East Asia, the risk of preterm delivery was almost double among those women diagnosed as having BV in early pregnancy (21%) compared to those who developed the condition in late pregnancy (11%)[21]. Using multiple logistic regression analysis, BV was found to be associated with a five-fold increased risk of preterm delivery when BV was detected before 16 weeks gestation independent of recognized risk factors such as previous preterm birth, black race and smoking[16]. Hay and co-workers also found a five-fold increased risk of late miscarriage in women with BV in early pregnancy[16] and women with recurrent second trimester miscarriages were far more likely to be colonized by BV than women with a history of recurrent first trimester miscarriages[22]. A number of studies have examined whether the detection and treatment of BV in pregnancy would reduce the morbidity and mortality associated with preterm delivery. Unfortunately these studies have varied in the choice of antibiotic, dose regime, route of administration, level of patient risk, gestational age at administration and success or failure, so no consensus has yet been reached about whether or not antibiotics are effective[23]. The effect of this treatment in our study on the outcome of pregnancy was the subject of a separate report[7] which showed a 60% statistically significant reduction in the incidence of preterm birth from 10% with placebo to 4% with CVC. There is logic in using intravaginal antibiotics for the treatment of BV in pregnancy since a large load of antibiotics is administered directly to where there is the heaviest colonization. There is also less likely to be unacceptable systemic side-effects.

Different bacterial species are distributed in different ways according to the different grades of Gram stain. *Mycoplasma hominis*, *Gardnerella vaginalis* and some anaerobes are only fully expressed in grade 3 Gram stain BV[24]. It may be that this represents a subgroup of women with BV in pregnancy that are most likely to benefit from CVC[25].

BV in pregnancy should be treated either to give symptomatic relief or following opportunistic screening of high risk women found to have BV to reduce the risks of adverse obstetric sequelae[5]. This study has shown that a 3-day course of CVC 2% is well tolerated and an effective way of treating BV in the second trimester of pregnancy.

Three other studies used CVC prophylactically[23,26,27] for the prevention of preterm birth without success but these have been criticized[28]. In one study[23] 100% of women were treated after 20 weeks gestation and in the other[26] 60% of women were treated after 20 weeks. In the study by Vermeulen[27] risk was based on previous preterm birth and the mean gestation at randomization was 20 weeks. This is in contrast to our published data[7] where all women were treated before 20 weeks gestation and 60% were treated before 16 weeks. We have also pointed out[29] that in the Vermeulen study, none of the seven women who delivered preterm had BV. This suggests other non-infectious pathology and a self-fulfilling prophesy that antibiotics would be unhelpful. In a subsequent report, Vermeulen and co-workers claimed that CVC, rather than preventing preterm birth, caused neonatal infectious morbidity[30]. We have pointed out the reason for this, that Vermeulen and colleagues are not eradicating abnormal colonization in early pregnancy, rather, they are decimating normal flora late in pregnancy[31]. We also pointed out that three of the references quoted in that paper about the use of CVC in pregnancy were erroneous. In all three studies pregnancy was a contraindication to study entry, in one of the studies recruitment was from a STI clinic and in another study 14% of the women had had a hysterectomy. We have received no response from the corresponding author.

### ACKNOWLEDGEMENTS

We are grateful to all the obstetricians at the three hospitals for allowing their patients to be admitted to the study. We are grateful to Dr F. E. Chisti, Wendy Davis and Jill Unerman for their hard work in the recruitment to and conduct of the study. We are also grateful to Pauline Mills for preparing the manuscript.

### REFERENCES

1. Nugent RP, Krohn MA, Hillier SL. Reliability of diagnosing bacterial vaginosis is improved by a standardized method of Gram-stain interpretation. *J Clin Microbiol* 1991;29:297–301
2. Hay PE, Taylor-Robinson D, Lamont RF. Diagnosis of bacterial vaginosis in a gynaecology clinic. *Br J Obstet Gynaecol* 1992;99:63–6
3. Amsel R, Totten PA, Spiegel CA, *et al*. Non-specific vaginitis. Diagnostic criteria and microbial and epidemiological associations. *Am J Med* 1993;74:14–22
4. Geary M, Lamont RF. Prediction of preterm birth. In Elder MG, Lamont RF, Romero R, eds. *Preterm Labor*. New York: Churchill Livingston, 51–63
5. Sexually transmitted disease treatment guidelines 2002. *Centers for Disease Control and Prevention*. Recomm Rep 2002 May 10;51:(No RR-6) 1–78
6. Thomason JL, Gelbart SM, Anderson RJ, *et al*. Statistical evaluation of diagnostic criteria for bacterial vaginosis. *Am J Obstet Gynecol* 1990;162: 155–60
7. Lamont RF, Duncan SLB, Mandal D, Bassett P. Intravaginal clindamycin to reduce preterm birth in women with abnormal genital tract flora. *Obstet Gynecol* 2003;101:516–22
8. Livengood CH, Thomason JL, Hill GB. Bacterial vaginosis: diagnostic and pathogenic findings during topical clindamycin therapy. *Am J Obstet Gynecol* 1990;163:515–21
9. Hillier S, Krohn MA, Watts DH, *et al*. Microbiologic efficacy of intravaginal clindamycin cream for the treatment of bacterial vaginosis. *Obstet Gynecol* 1990;76:407–13
10. Borin MT, Powley GW, Tackwell KR, Batts DH. Absorption of clindamycin in bacterial vaginosis patients after intravaginal application of clindamycin phosphate 2% cream. *Clin Pharmacol Ther* 1992;51:185
11. Powley GW, Timm JA, Le VH. Clindamycin vaginal cream versus placebo vaginal cream for bacterial vaginosis during pregnancy. (Protocol M/1115/0006). *Upjohn Technical Report* 9156-92-019 December 1992. (Held on file Pharmacia and Upjohn, Kalamazoo, USA)
12. Powley GW, Timm JA, Greenwald CA. Comparison of two dosing regimens of 2% clindamycin vaginal cream for the treatment of bacterial vaginosis – 3 days versus 7 days. (Protocol M/1115/0020). *Upjohn Technical Report* 9156-93-006 December 1993. (Held on file at Pharmacia and Upjohn, Kalamazoo, USA)
13. Ahmed-Jusuf IH, Shahmanesh M, Arya OP. The treatment of bacterial vaginosis with a three day course of 2% clindamycin cream: results of a multicentre, double blind, placebo controlled trial. *Genitourin Med* 1995;71:254–6
14. Lamont RF. Bacterial vaginosis. In Studd JWW, Jardine-Brown C, eds. *The Yearbook of the RCOG*, London: Parthenon Publishing, 1995:149–60
15. Kent HL. Epidemiology of vaginitis. *Am J Obstet Gynecol* 1991;165:1168–76
16. Hay PE, Lamont RF, Taylor-Robinson D, *et al*. Abnormal bacterial colonisation of the genital tract as a marker for subsequent preterm delivery and late miscarriage. *Br J Med* 1994;308:295–8
17. Newton ER, Prihoda TK, Gibbs RS. A clinical and microbiologic analysis of risk factors for puerperal endometritis. *Obstet Gynecol* 1990;75: 402–6
18. Gibbs RS. Chorioamnionitis and bacterial vaginosis. *Am J Obstet Gynecol* 1993;169:460–2
19. Chin BM, Lamont RF. The microbiology of preterm labor and delivery. *Cont Rev Obstet Gynecol* 1997;9:285–96
20. Kurki T, Sivonen A, Renkonen OV, *et al*. Bacterial vaginosis in early pregnancy and pregnancy outcome. *Obstet Gynecol* 1992;80:173–7
21. Riduan JM, Hillier SL, Utomo B, *et al*. BV and prematurity in Indonesia: association in early and late pregnancy. *Am J Obstet Gynecol* 1993; 169:175–8
22. Llahi-Camp JM, Rai R, Ison C, *et al*. Association of bacterial vaginosis with a history of second trimester miscarriage. *Hum Reprod* 1996;11: 11575–8
23. McGregor JA, French JI, Jones W, *et al*. Bacterial vaginosis is associated with prematurity and vaginal fluid mucinase and sialidase: results of a controlled trial of topical clindamycin cream. *Am J Obstet Gynecol* 1994;170:1048–60
24. Rosenstein IJ, Morgan DJ, Sheehan M, *et al*. Bacterial vaginosis in pregnancy: distribution of bacterial species in different gram-stain categories of the vaginal flora. *J Med Microbiol* 1996;45:120–6
25. Rosenstein IJ, Morgan DJ, Lamont RF, *et al*. Effect of vaginally applied clindamycin on vaginal microbial flora and outcome of pregnancy in women with bacterial vaginosis. *Infect Dis Obstet Gynecol* 2000;8:158–65
26. Joesoef MR, Hillier SL, Wiknjosastro G, *et al*. Intravaginal clindamycin treatment for bacterial vaginosis: effects on preterm delivery and low birth weight. *Am J Obstet Gynecol* 1995;173: 1527–31
27. Vermeulen GM, Bruinse HW. Prophylactic administration of clindamycin 2% vaginal cream to reduce the incidence of spontaneous preterm birth in women with an increased recurrence risk: a randomized placebo controlled double blind trial. *Br J Obstet Gynaecol* 1999;106:652–7
28. Lamont RF. Antibiotics for the prevention of preterm birth. *N Engl J Med* 2000;342:581–3
29. Mason MR, Adinkra PE, Lamont RF. Prophylactic administration of clindamycin 2% vaginal cream to reduce the incidence of spontaneous preterm birth in women with an increased risk: a randomized placebo controlled double blind trial. *Br J Obstet Gynaecol* 2000;170:295–6;letter
30. Vermeulen GM. Changes in vaginal flora after 2% clindamycin vaginal cream in women at high risk of preterm birth. *Br J Obstet Gynaecol* 2001; 108:697–700
31. Lamont RF. Changes in vaginal flora after 2% clindamycin vaginal cream in women at high risk of preterm birth. *Br J Obstet Gynaecol* (in press)

Received 10/29/02; Accepted 07/16/03
